# The integrated multiomic diagnosis of sporadic meningiomas: a review of its clinical implications

**DOI:** 10.1007/s11060-021-03874-9

**Published:** 2021-11-30

**Authors:** Stephanie M. Robert, Shaurey Vetsa, Arushii Nadar, Sagar Vasandani, Mark W. Youngblood, Evan Gorelick, Lan Jin, Neelan Marianayagam, E Zeynep Erson-Omay, Murat Günel, Jennifer Moliterno

**Affiliations:** 1 Department of Neurosurgery, Yale School of Medicine, 15 York St, LLCI 810, New Haven, CT 06520-8082 USA; 2 The Chenevert Family Brain Tumor Center, Smilow Cancer Hospital, New Haven, CT USA; 3 Department of Neurological Surgery, Northwestern University, Chicago, IL USA

**Keywords:** Meningioma, Sporadic, Genomic subgroups, Precision medicine

## Abstract

**Introduction:**

Meningiomas are generally considered “benign,” however, these tumors can demonstrate variability in behavior and a surprising aggressiveness with elevated rates of recurrence. The advancement of next-generation molecular technologies have led to the understanding of the genomic and epigenomic landscape of meningiomas and more recent correlations with clinical characteristics and behavior.

**Methods:**

Based on a thorough review of recent peer-reviewed publications (PubMed) and edited texts, we provide a molecular overview of meningiomas with a focus on relevant clinical implications.

**Results:**

The identification of specific somatic driver mutations has led to the classification of several major genomic subgroups, which account for more than 80% of sporadic meningiomas, and can be distinguished using noninvasive clinical variables to help guide management decisions. Other somatic genomic modifications, including non-coding alterations and copy number variations, have also been correlated with tumor characteristics. Furthermore, epigenomic modifications in meningiomas have recently been described, with DNA methylation being the most widely studied and potentially most clinically relevant. Based on these molecular insights, several clinical trials are currently underway in an effort to establish effective medical therapeutic options for meningioma.

**Conclusion:**

As we enhance our multiomic understanding of meningiomas, our ability to care for patients with these tumors will continue to improve. Further biological insights will lead to additional progress in precision medicine for meningiomas.

## Introduction

Meningiomas are the most common primary central nervous system (CNS) tumor, accounting for ~ 40% of intracranial tumors and 54% of nonmalignant tumors [1, 2]. While the majority are slow growing, when intervention is necessary due to size and/or symptomatology, treatment involves neurosurgical resection. Radiotherapy is used more variably and despite significant advancements in our understanding of meningioma biology, there remains no effective pharmacological therapies.

Typically considered “benign,” meningiomas are defined by location and histology. Classified based on atypical features, including mitoses and brain invasion, they are divided into three World Health Organization (WHO) grades. Grade I (~ 80% of meningiomas) demonstrate low risk of recurrence; grade II (atypical) and grade III (anaplastic) exhibit higher rates of growth, with the latter being frankly “malignant” and demonstrating recurrence rates > 70% after gross total resection [3]. There are 15 histological subtypes, with nine variants recognized in grade I tumors [4]. These tumors do not, however, always behave according to their WHO grading, with reports of histologically-confirmed grade I tumors demonstrating unexpected recurrence, malignant transformation, and aggressive behavior [5], underscoring their complexity and heterogeneity.

The last decade has provided insight into meningioma biology, particularly for those arising sporadically. Several recently identified somatic driver mutations have defined new clinically-relevant molecular subgroups [6] and this knowledge has shifted focus to integrated diagnosis, targeted treatment, and novel opportunities for pharmacological development. Importantly, the WHO grading scale has been updated to incorporate molecular diagnostics (see below) [7]. In this review, we discuss our current understanding of the multiomic landscape of meningiomas, with a focus on relevant clinical implications.

## Genomic landscape of sporadic meningiomas

Germline mutations are well-established drivers of meningioma formation, especially those arising in the context of genetic diseases such as neurofibromatosis type II (NF2), Gorlin and Cowden syndromes [8]. Recent focus has turned to characterization of sporadic meningiomas, which account for the majority of these tumors and typically occur without a clear inciting event. Importantly, meningiomas are unique in harboring a small number of somatic mutations, and this “cleaner” genomic architecture has allowed expeditious insight into the pathophysiology of sporadic meningiomas.

### Genomic subgroup classification based on key driver mutations

**Fig. 1:**
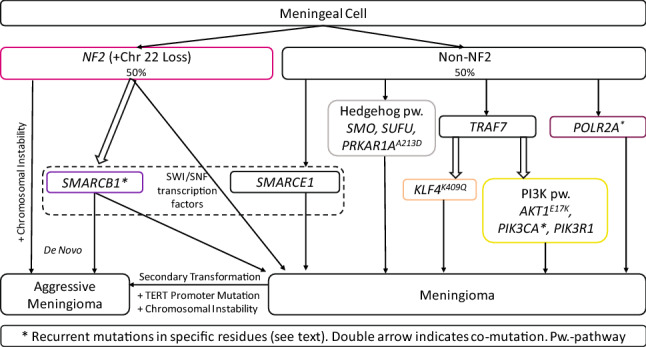
Somatic mutational profile of sporadic meningiomas

Major genomic subgroups have been defined based on specific somatic driver mutations in ~ 80% of sporadic meningiomas [9]. These include (1) *Neurofibromatosis-2* (*NF2*) with or without co-mutation in *SWI/SNF Related, Matrix Associated, Actin Dependent Regulator of Chromatin, Subfamily B, Member 1* (*SMARCB1*); (2) mutations in the WD40 region of *TNF Receptor-Associated Factor 7 (TRAF7*), which can occur alone or (3) with a recurrent mutation in *Kruppel-Like Factor 4* (*KLF4*^*K409Q*^) or (4) mutations in PI3K (phosphoinositide 3-kinase) pathway molecules, including *PIK3CA*, *PIK3R1*, and *AKT1*^*E17K*^; (5) Hedgehog (HH) signaling molecules (i.e. *SMO*, *SUFU*, *PRKAR1A*); (6) recurrent mutations in *RNA Polymerase II Subunit A* (*POLR2A*^*Q403K*^ or ^*L438_H439del*^), or (7) *SMARCE1* mutations [6, 9–11]. Over half of sporadic meningiomas harbor somatic *NF2*-mutations and noninvasive clinical diagnostics can differentiate between *NF2*-mutated and non-mutated meningiomas, with important implications for patient care [6]. These genomic subgroup classifications, important somatic driver mutations, and their relationships are illustrated in Fig. 1.

### NF2-mutated meningiomas

The most common driver mutation is biallelic loss of *NF2*, accounting for > 50% of meningiomas [12]. A tumor suppressor, *NF2* encodes a cytoskeleton scaffold protein involved in cell proliferation and apoptosis. Aggressive *NF2*-mutated meningiomas acquire chromosomal instability or co-mutation in another tumor suppressor gene, *SMARCB1* [13]. Most somatic *NF2* mutations are in solitary, sporadic meningiomas, however, they can occur in radiation-induced and multiple meningiomas [14].

**Fig. 2:**
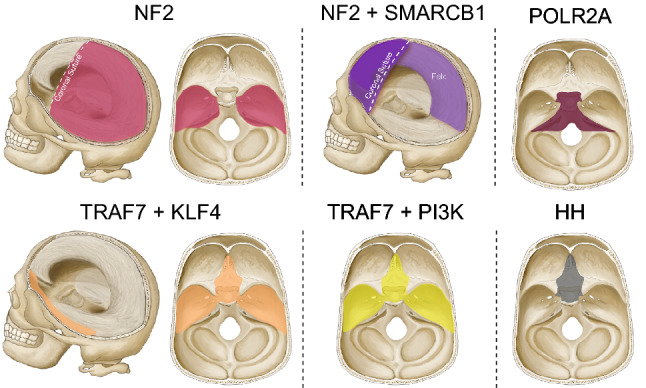
Localization of molecular subtypes to unique brain regions. Adapted from Youngblood et al., 2019 with permission from the Journal of Neurosurgery [6]

*NF2*-mutated meningiomas demonstrate larger volumes, fibrous or atypical histology, male predominance, and preferentially occur along the cerebral convexities posterior to the coronal suture (Fig. 2) [6, 15]. Along the skull base, *NF2*-mutant meningiomas show lateral to medial gradient, originating along the lateral sphenoid wing, invading the bone [16]. These mutations are associated with preoperative seizures, predict a more aggressive clinical course, shorter progression free survival [17], and correlate with higher proliferation indexes [6]. They are enriched in recurrent tumors, especially when present with recurrent *SMARCB1* mutations. *SMARCB1,* part of the switch/sucrose non-fermentable (SWI/SNF) chromatin-remodeling complex protein [18], demonstrates recurrent p.Arg383Gln or p.Arg386His mutations [18–20]. Unlike *NF2*-mutated meningiomas, *SMARCB1/NF2* co-mutations localize anterior to the coronal suture and medially along the falx [18].

### Non-NF2 mutated meningiomas

*TRAF7* is the most common somatic mutation in non-*NF2* meningiomas, present in ~ 25% of sporadic tumors [10]. As a proapoptotic E3 ubiquitin ligase, *TRAF7* activates downstream signaling pathways including mitogen-activated protein kinase (MAPK) and nuclear factor-kB (NF-kB), with mutations causing unregulated NF-kB activity and cell proliferation [10, 13]. *TRAF7* tumors demonstrate meningothelial histology and higher-grade characteristics [6, 18]. Mutations in the *KLF4* or PI3K pathway, such as *AKT1* or *PIK3CA*, co-occur with *TRAF7*. Mutations in *KLF4,* a zinc finger transcription factor, affect the specific lysine residue at position 409 (*KLF4*^*K409Q*^) [10, 21]. *TRAF*/*KLF4*^*K409*^ mutations are found in ~ 10% of meningiomas, are typically located along the lateral skull base (Fig. 2), demonstrate secretory histology, and cause significant peritumoral edema [6, 10]. *TRAF7* with co-mutations in PI3K/AKT pathway represent an additional ~ 10%, and localize along the sphenoid wing and medial anterior skull base [11]. Most common is a recurrent *AKT1*^*E17K*^ mutation, resulting in constitutive activation of PI3K/mTOR (mammalian target of rapamycin) pathway and upregulation of growth factor-induced cellular survival [6]. *PIK3CA*, *PIK3R1*, and *AKT3* mutations similarly increase PI3K/AKT signaling and are found in ~ 20% of anterior skull base or convexity meningiomas (Fig. 2) [6, 9]. Recent data demonstrates that sphenoid wing meningiomas causing hyperostosis are associated with *TRAF7* variants [16].

Hedgehog (HH) is another important signaling pathway in meningioma formation. Proteins in this pathway direct developmental patterning and cell differentiation during embryogenesis, and in adult tissues, regulate stem-cell mediated cell cycle activation and tissue regeneration [10, 11]. Mutations *Smoothened* (*SMO*) and *Suppressor of Fused* (*SUFU*) genes are the most common, are associated with meningothelial and low grade histology [22], and occur in the anterior skull base, typically midline along the olfactory groove or planum sphenoidale; ~ 45% of midline meningiomas demonstrate HH pathway mutations [23]. Given HH pathway’s role in embryonic midline patterning, these findings underscore the importance of developmental and regulatory gene dysfunction in meningiogenesis.

Recurrent somatic mutations in *POLR2A* are found in ~ 6% of meningiomas [9]. *POLR2A* encodes RPB1, a member of the polymerase II complex involved in mRNA transcription. Mutations in this enzyme are rare and meningiomas are the only pathology known to harbor these recurrent mutations [9]. *POLR2A* mutations are thought to alter expression of meningeal progenitor and differentiation genes, including Zic family members 1 and 4 (*ZIC1/ZIC4*) and Wnt family protein 6 and 10A (*WNT6/WNT10A*) [9]. These tumors are grade I, demonstrate female predominance, meningothelial histology, and occur in the sella, clivus and posterior fossa [6, 9] (Fig. 2).

### Other somatic mutations

Mutations in *SMARCE1*, another member of the SWI/SNF complex, have been described in higher-grade tumors. *SMARCE1* loss is associated with clear cell meningiomas, WHO grade II tumors with elevated recurrence [19, 24]. Additional mutations have been described, includin*g ARID1A* and *SMARCA4*, with *ARID1A* mutations being present in ~ 20% of grade I and II tumors, 15% of grade III meningiomas, and associated with increased mortality [25].

### Somatic, non-coding genomic alterations

As next generation sequencing techniques advance, we are learning the importance of non-coding regions of the genome. The best understood is telomerase reverse transcriptase *(TERT)* promoter mutations. These mutations, found in ~ 6% of meningiomas, cause *TERT* upregulation, which abnormally extends the telomeres of meningioma cells, enhancing their lifespan [26]. *TERT* promoter mutations are important in atypical transformation of Grade 1 meningiomas, and are thus found in higher grade, malignant tumors, predicting increased recurrence risk [26].

## Somatic copy-number variations

Copy number variations (CNV), including simple duplications, deletions, or complex rearrangements of genomic regions result in altered transcriptional regulation; tumorigenic CNVs cause deletion of tumor suppressor genes or amplification of oncogenes. The most frequent CNV in sporadic meningiomas is chromosome 22q deletion, which contains *NF2* and *SMARCB1*, among others [15, 27]. Abnormalities involving Chromosome 1 are also common, with loss of the short arm (1p) being the second most common chromosomal abnormality, found in 70–80% of atypical meningiomas [28]. In grade I tumors, chromosome 1p deletion increases the risk of recurrence [29] and 1q gain is associated with atypical tumors and shorter progression-free survival [28].

Other frequent CNVs include chromosome 9p deletion, which contains tumor suppressor genes *Cyclin-Dependent Kinase Inhibitor 2A* and *2B* (*CDKN2A/B*) [15], and loss of chromosomes 2p, 4p, 6q, 7p, 9p, 10q, 11p, 14q, 17q, and 18q [29–37]. Chromosome 2p loss is associated with choroid meningiomas, while anaplastic meningiomas demonstrate chromosome 1p, 6p, 14, and 22q losses [15]. *CDKN2A/B* mutations are associated with recurrent and aggressive tumors [38, 39], multiple copies of chromosome 5 are found in angiomatous meningiomas [36], gains in chromosome 22q are more often found in skull base regions, while losses at 1p, 8p, 14q and 22q correlate with non-skull base locations [33, 40].

Although studies have suggested that grade II/III lesions have more numerous and larger CNVs [29, 33], a recent study demonstrated that while de novo atypical *NF2* tumors have more large-scale CNVs, there was no difference between non-*NF2* meningioma grades [20]. Chromosome 14q loss showed the largest difference between atypical and low grade *NF2*-mutated meningiomas [20]. Furthermore, loss of chromosomes 1, 4p, 10p, 14, and 22, and higher numbers of cumulative CNVs are more common in recurrence [33] (Table 1).

**Table 1:** CNV Associations with meningioma characteristics

CNV		Known characteristics	Refs.
1p	L	High grade, increased recurrence, non-skull-base location, larger volume	[30, 33]
4p	L	Increased recurrence	[33]
4q	L	High grade	[37]
6q	L	High grade, increased recurrence	[32, 37]
8p	L	Non-skull-base location	[33]
9p	L	High grade, increased recurrence	[30, 33]
10p	L	High grade, increased recurrence	[31, 33]
10q	L	High grade	[31]
11p	L	Increased recurrence	[33]
14q	L	High grade, increased recurrence, non-skull-base location	[32, 79]
17q	L	Increased recurrence	[34]
18q	L	High grade, increased recurrence	[32, 79]
22q	L	High grade, increased recurrence, non-skull-base location	[6, 36]
X-Chr	L	High grade	[35]
Y-Chr	L	High grade	[35]
1q	G	High grade	[79]
5	G	Angiomatous Histology	[36]
9q	G	High grade	[79]
12q	G	High grade	[79]
15q	G	High grade	[79]
17q	G	High grade	[79]
20q	G	High grade	[79]
22p	G	Skull-base location	[33]
22q	G	Skull-base location	[33]
17q	A	High grade	[30]

*G* gain, *A* amplification, *L* loss

## Epigenomic modifications

The role of epigenomic changes such as aberrant DNA methylation, histone methylation, and acetylation is also being defined [41] (Table 2), with DNA methylation being most well-understood. Hypermethylation of DNA regulatory regions, leading to gene silencing, correlates with tumor aggressiveness and recurrence [20, 42]. Homeobox family genes (HOXA) [43] are often hypermethylated in aggressive tumors, and tumors with a group of five HOXA genes (*HOXA6, HOXA9, PENK, UPK3A,* and *IGF2BP1*) hypermethylated together, exhibit higher recurrence rates [44]. Similarly, the genomic locus harboring *hypoxia inducible factor* (*HIF*), which inhibits proliferation through angiogenesis regulation, demonstrates increased methylation [45], possibly contributing to tumor growth. Although clear relationships between genomic mutations and epigenomic regulation has yet to be defined [19, 41], evidence points to important interplays. A recent study investigating the epigenomics of clear cell meningiomas found a unique *SMARCE1* mutation signature [19], bridging mutations in *SMARCE1* to the observed epigenomic changes. Hypomethylation has also been associated with increased aggressiveness of some tumors [46].

**Table 2:** Epigenetic changes and associated clinical characteristics in meningioma

Epigenetic alteration	Associated meningioma characteristics	Genes	Refs.
Hypermethylation, promoter region	Tumor recurrence	CDH13, MLH1, NDRG2, RASSF1A, CGTF, HOXA genes	[42, 66–68]
	Tumor progression	TP73, TP53, CDKN2A, CDKN2B, CDKN2C, ADAM23, RB1, DAPK1, VHL, ER, RUNX3, DCL1, HIF, WNK2, NDRG2, HOXA genes, TIMP3, FOXM1 Inhibitors	[42, 47, 66, 67]
	Angiogenesis	HIF, THBS1	[42, 66, 67]
	Higher grade	MAL2, RASSF1A, IGF2BP1, PDCD1, Aberrant CpG Islands, DNMT-3B, GSTP1	[42, 67–69]
	Longer survival	MGMT	[42]
Dysregulation	Higher grade	UPA, ALPL	[42]
	Tumor recurrence	TMEM30B, TGFBeta—LMO4,	[66]
	Tumor progression	CTNNB1, ALPL, IGFBP3, NOTCH	[66]
Abnormal LNCRNA expression	Tumor progression	LINC00702, SNHG1, Downregulation MEG3, LINC00460	[70–73]
	Tumor recurrence	HIST1H1C	[66]
	Higher grade	LINC00702	[71]
Abnormal miRNA expression	Tumor progression	miR-29c-3p, miR-190a, miR-21, miR-335	[42, 67, 74]
	Recurrence	miR-190a, Downregulation of miR-219c-5, miR-96-5p, miRNA-224	[42, 74]
	Higher grade	Downregulated miR-145, miR218, miR-34a, miRNA-224	[42, 67]
	Lower grade	miR-107	[42]
Histone modification	Recurrence	Loss of trimethylation of lysine 27 of Histone3	[72]
	Worse outcome	Loss of trimethylation of lysine 27 of Histone3	[75]
	Tumor progression	NAT2 acetyltransferase, EZH2 downregulation	[69, 76]
	Disturbed chromatin regulation in grade I and grade 3 tumors	KDM5C	[67, 77]
	Disturbed chromatin regulation in grade 2	KDM6A	[67, 77]
Abnormal SnoRNA expression	Higher grade	SNORA46, SNORA48	[78]
	Tumor progression	SNORD50A	[78]

DNA methylation patterns can differentiate *NF2*-mutant from non-*NF2* mutant tumors, with further differentiation of non-*NF2* mutant tumors into atypical versus “benign.” In de novo formation of atypical *NF2*-mutant meningiomas, hypermethylation is either associated with large-scale CNVs, or gained due to *SMARCB1* co-mutations [20]. Separately, “high” methylation levels were found in tumors from older patients, those with increased somatic mutation burden, higher tumor grade, convexity location, and *NF2* mutations [47]. *FOXM1* expression, of which *NF2* is a negative regulator, is increased with FOXM1/Wnt signaling pathway activation, which likely underlies enhanced cell proliferation [47].

Histone modification and microRNAs (miRNAs) are important in meningioma pathophysiology. Trimethylation of lysine 27 of histone H3 (H3K27me3) predicts worse outcomes and faster recurrence for grade I/II meningiomas [48]. EZH2, the catalytic subunit of the Polycomb Repressive Complex 2 (PRC2), is thought to mediate this effect by causing long-term gene expression silencing. Using ChIP-seq, we demonstrated H3K27me3 profiles differentiate atypical and benign meningiomas, with increased H3K27me3 signal in atypical tumors [20]. In a separate study, *NF2* and *SUFU* mutations were found enriched among tumors lacking H3K27me3 [49]. miRNA expression levels differ between tumor and normal cells, and between atypical and anaplastic meningiomas [50]. For example, miRNA-107 acts as a tumor suppressor and is decreased in higher grade lesions, while miRNA-21 acts as an anti-apoptotic factor and its expression is reported to be increased in meningiomas [51].

## Pathways to malignancy

Genetic markers of meningioma aggressiveness is an active area of investigation, aiming to identify molecular signatures for patients at risk of transformation. Unique mutations are being discovered in rarer, more malignant subsets of meningiomas. *TERT* promoter mutations are associated with recurrence as well as progression to higher grades [52]. The *breast cancer (BRCA)1-associated protein-1 tumor suppressor* gene (*BAP1*) has been linked to a clinically aggressive rhabdoid subtype [53] and both germline and somatic mutations in *BAP1* predict faster recurrence [54]. As discussed above, DNA methylation correlates with  aggressiveness and H3K27me3 loss identifies a subset of grade 2 meningiomas with increased recurrence risk [48]. Gene co-expression analysis is also being used to predict tumor behavior using meta-gene markers. One recent co-expression module identified, E2F4/FOXM1, predicts increased meningioma aggressiveness [55], correlating with previous identification of *FOXM1* and *E2F2* expression networks activated in atypical meningiomas [20]. Further insight into genetic alterations and gene co-expression networks will improve our ability to predict tumor behavior.

## Integrated diagnosis

The newly released WHO Classification of Tumors of the Central Nervous System 2021 updated meningioma grading parameters, including introducing several of these biomarkers. Meningiomas remain a single entity with 15 subtypes; however, criteria defining atypical (grade II) or anaplastic (grade III) are applied regardless of subtype. Subtypes with higher recurrence rates, such as chordoid and clear cell, are considered grade 2 tumors; however, note is made of the need for improved understanding of prognostic markers for these atypical meningiomas. Mutations in *SMARCE1*, *BAP1*, *KLF4*/*TRAF7*, *TERT* promoter, and *CDKN2A/B* deletion, H3K27me3 loss and methylation profiling are now officially associated with the classification and grading of meningiomas [7].

## Clinical & treatment implications

### Guidance of patient management decisions

Management of meningiomas is typically based on location and symptomatology. For small, asymptomatic tumors, patients can be followed with imaging to monitor growth. Larger and/or symptomatic lesions can undergo surgical resection, with possible adjuvant radiotherapy. Prediction of tumor behavior through genomic mutation analysis is beginning to guide clinical decisions. Increasingly, predictive scores are being developed based on integrated transcriptome data [56], and grading systems are being proposed based on epigenomic modifications and chromosomal variations [33, 57]. One DNA methylation-based classification identified low grade, “benign” tumors, with high, rapid recurrence risk, and conversely, higher grade tumors with lower risk. Further, unsupervised clustering differentiated meningiomas from other extra-axial tumors, and determined distinct, clinically-relevant classes predicting recurrence [57]. Another study using DNA methylation profiles developed a methylome-based five-year recurrence-free survival prediction model demonstrating improved recurrence predictions compared to clinical or pathology grading-based systems [58]. Driver et al. developed a three-tiered integrated grading (grades 1–3) incorporating mitotic count and loss of chromosomes 1p, 3p, 4, 6, 10, 14q, 18, 19, or *CDKN2A*, and demonstrated that this grading scheme improves the current WHO system in predicting progression-free survival [59]. Furthermore, Patel et al. identified three groups using RNA-sequencing and whole-exome sequencing that correlate with recurrence [60]. Using a multi-omic approach, Nassiri et al. defined integrative molecular groups through combined analysis of DNA somatic copy-number aberrations, DNA somatic point mutations, DNA methylation, and messenger RNA abundance. They identified four molecular groups that independently associated with recurrence-free survival and better predicted time to recurrence than WHO grading [61].

Importantly, genomic characterization informs preoperative and postoperative decision-making through insights about potential tumor behavior and recurrence risk. Our recent work identified aggressive genomic subgroups with mutations in *NF2*, *TRAF7,* and HH and PI3K pathways, are associated with a ~ 22 times higher two-year recurrence rate [62]. Separately, chromosome 1p deletion and *CDKN2A/B* loss are independently associated with early recurrence and higher grade [29, 39]. Therefore, a convexity meningioma, for instance, occuring in a low surgical risk location may be considered for earlier resection, given they are often *NF2*-mutated and demonstrate higher grade, atypical features. Those arising in surgically challenging skull base locations may be considered for close surveillance and debulking, with consideration of earlier adjuvant radiation if confirmed to be an aggressive subtype.

Management of residual and recurrent lesions remains debated, with those demonstrating higher risk typically undergoing radiation and/or repeat surgical resection [63]. However, limits to the number of resections and radiation treatments patients can safely undergo necessitates more effective therapeutic options. Identification of unique driver mutations and advancements in molecular profiling are opening new avenues for targeted, pharmaceutical therapies and allowing implementation of patient-centered treatment protocols.

### Development of targeted therapies and precision oncological care for meningioma

Multiple clinical trials targeting genetic mutations are underway. One promising target is the mTOR pathway, of which *NF2* is a negative regulator. Studies investigating mTOR inhibition using Everolimus (NCT01880749, NCT01419639), Everolimus + Octreotide (NCT02333565), or Vistusertib (AZD2014) (NCT03071874, NCT02831257), are showing promising results in progression free survival and tumor volume [64]. Vismodegib (SMO inhibitor) and GSK2256098 (FAK inhibitor) are being used in meningiomas with HH pathway (SMO) or NF2 pathway (FAK) mutations, respectively (NCT02523014).

A phase I study of Alpelisib and Trametinib, PI3K and MEK inhibitors, respectively (NCT03631953), via a combinational approach (inclusion criteria does not include genomic events associated with PI3K activation) in aggressive and recurrent meningiomas is underway. Ribociclib, a cyclin-dependent kinase (CDK) inhibitor, targeting chromosomal abnormalities in the p53/pRB pathway, which is increased in higher grade tumors, is being used in grade II–III meningiomas (NCT02933736) [65]. In addition, clinical trials are investigating the role of radiotherapy, immunotherapy, or a combination of these therapies as primary and adjuvant treatment of meningiomas (see “Appendix” for NCT numbers).

In addition to developing new pharmacological targets, genomic characterization of meningiomas is improving our diagnostic capabilities. Youngblood, et al. used machine learning algorithms to predict underlying genomic events (*NF2* vs. non-*NF2*) based on clinical and imaging features [6]. The ability to use non-invasive analysis to predict tumor genetics, and therefore behavior, will provide invaluable phenotypic meningioma profiles to guide treatment and management decisions without invasive procedures.

## Conclusions

Though meningiomas are frequently considered “benign,” morbidity and therapeutic challenges are often encountered, calling this classification into question. As we continue to develop a deeper understanding of the genomic and epigenomic landscape of meningiomas, associated pathophysiological mechanisms suggest a more heterogeneous group than initially thought, with variable clinical behavior, aggressiveness, and recurrence rates. Incorporating genomic and molecular features with histopathological characteristics is improving our diagnostic accuracy and suggesting new, targetable pathways for pharmaceutical interventions. As our understanding of meningioma genomics deepens, our ability to further personalize medical and surgical management of patients with meningiomas will continue to improve, as will our ability to offer targeted therapies and advanced precision oncological care.

## Appendix

*Radiotherapy Clinical Trials* NCT01166321, NCT02978677, NCT03180268, NCT01795300, NCT02693990, NCT01117844, NCT04127760, NCT00626730, NCT00895622, NCT02594709, NCT04278118, NCT03604978, NCT03267836, NCT04659811.

*Iimmunotherapy Clinical Trials* NCT02648997, NCT03279692, NCT04728568, NCT04501705, NCT03016091, NCT03604978, NCT03267836, NCT04659811.

## References

[1] Miller KD, Ostrom QT, Kruchko C, Patil N, Tihan T, Cioffi G, Fuchs HE, Waite KA, Jemal A, Siegel RL, Barnholtz-Sloan JS (2021). Brain and other central nervous system tumor statistics, 2021. CA Cancer J Clin.

[2] Ostrom QT, Patil N, Cioffi G, Waite K, Kruchko C, Barnholtz-Sloan JS (2020). CBTRUS statistical report: primary brain and other central nervous system tumors diagnosed in the United States in 2013–2017. Neuro Oncol.

[3] Maier H, Ofner D, Hittmair A, Kitz K, Budka H (1992). Classic, atypical, and anaplastic meningioma: three histopathological subtypes of clinical relevance. J Neurosurg.

[4] Louis DN, Perry A, Reifenberger G, Von Deimling A, Figarella-Branger D, Cavenee WK, Ohgaki H, Wiestler OD, Kleihues P, Ellison DW (2016). The 2016 World Health Organization classification of tumors of the central nervous system: a summary. Acta Neuropathol.

[5] Corniola MV, Lemee JM, Meling TR (2020). Histological transformation in recurrent WHO grade I meningiomas. Sci Rep.

[6] Youngblood MW, Duran D, Montejo JD, Li C, Omay SB, Ozduman K, Sheth AH, Zhao AY, Tyrtova E, Miyagishima DF, Fomchenko EI, Hong CS, Clark VE, Riche M, Peyre M, Boetto J, Sohrabi S, Koljaka S, Baranoski JF, Knight J, Zhu H, Pamir MN, Avsar T, Kilic T, Schramm J, Timmer M, Goldbrunner R, Gong Y, Bayri Y, Amankulor N, Hamilton RL, Bilguvar K, Tikhonova I, Tomak PR, Huttner A, Simon M, Krischek B, Kalamarides M, Erson-Omay EZ, Moliterno J, Gunel M (2019). Correlations between genomic subgroup and clinical features in a cohort of more than 3000 meningiomas. J Neurosurg.

[7] Louis DN, Perry A, Wesseling P, Brat DJ, Cree IA, Figarella-Branger D, Hawkins C, Ng HK, Pfister SM, Reifenberger G, Soffietti R, von Deimling A, Ellison DW (2021). The 2021 WHO Classification of Tumors of the Central Nervous System: a summary. Neuro Oncol.

[8] Kerr K, Qualmann K, Esquenazi Y, Hagan J, Kim DH (2018). Familial syndromes involving meningiomas provide mechanistic insight into sporadic disease. Neurosurgery.

[9] Clark VE, Harmanci AS, Bai H, Youngblood MW, Lee TI, Baranoski JF, Ercan-Sencicek AG, Abraham BJ, Weintraub AS, Hnisz D, Simon M, Krischek B, Erson-Omay EZ, Henegariu O, Carrion-Grant G, Mishra-Gorur K, Duran D, Goldmann JE, Schramm J, Goldbrunner R, Piepmeier JM, Vortmeyer AO, Gunel JM, Bilguvar K, Yasuno K, Young RA, Gunel M (2016). Recurrent somatic mutations in POLR2A define a distinct subset of meningiomas. Nat Genet.

[10] Clark VE, Erson-Omay EZ, Serin A, Yin J, Cotney J, Ozduman K, Avsar T, Li J, Murray PB, Henegariu O, Yilmaz S, Gunel JM, Carrion-Grant G, Yilmaz B, Grady C, Tanrikulu B, Bakircioglu M, Kaymakcalan H, Caglayan AO, Sencar L, Ceyhun E, Atik AF, Bayri Y, Bai H, Kolb LE, Hebert RM, Omay SB, Mishra-Gorur K, Choi M, Overton JD, Holland EC, Mane S, State MW, Bilguvar K, Baehring JM, Gutin PH, Piepmeier JM, Vortmeyer A, Brennan CW, Pamir MN, Kilic T, Lifton RP, Noonan JP, Yasuno K, Gunel M (2013). Genomic analysis of non-NF2 meningiomas reveals mutations in TRAF7, KLF4, AKT1, and SMO. Science.

[11] Brastianos PK, Horowitz PM, Santagata S, Jones RT, McKenna A, Getz G, Ligon KL, Palescandolo E, Van Hummelen P, Ducar MD (2013). Genomic sequencing of meningiomas identifies oncogenic SMO and AKT1 mutations. Nat Genet.

[12] Ruttledge MH, Sarrazin J, Rangaratnam S, Phelan CM, Twist E, Merel P, Delattre O, Thomas G, Nordenskjold M, Collins VP (1994). Evidence for the complete inactivation of the NF2 gene in the majority of sporadic meningiomas. Nat Genet.

[13] Nazem AA, Ruzevick J, Ferreira MJ (2020). Advances in meningioma genomics, proteomics, and epigenetics: insights into biomarker identification and targeted therapies. Oncotarget.

[14] Agnihotri S, Suppiah S, Tonge PD, Jalali S, Danesh A, Bruce JP, Mamatjan Y, Klironomos G, Gonen L, Au K (2017). Therapeutic radiation for childhood cancer drives structural aberrations of NF2 in meningiomas. Nat Commun.

[15] Zhao AY, Youngblood MW, Erson-Omay EZ, Moliterno J, Gunel M (2020) The genomic landscape of meningiomas. In: Meningiomas. Springer, pp 35–55

[16] Jin L, Youngblood MW, Gupte TP, Vetsa S, Nadar A, Barak T, Yalcin K, Aguilera SM, Mishra-Gorur K, Blondin NA, Gorelick E, Omay SB, Pointdujour-Lim R, Judson BL, Alperovich M, Aboian MS, McGuone D, Gunel M, Erson-Omay Z, Fulbright RK, Moliterno J (2021). Type of bony involvement predicts genomic subgroup in sphenoid wing meningiomas. J Neurooncol.

[17] Gupte TP, Li C, Jin L, Yalcin K, Youngblood MW, Miyagishima DF, Mishra-Gorur K, Zhao AY, Antonios J, Huttner A, McGuone D, Blondin NA, Contessa JN, Zhang Y, Fulbright RK, Gunel M, Erson-Omay Z, Moliterno J (2020). Clinical and genomic factors associated with seizures in meningiomas. J Neurosurg.

[18] Smith MJ (2015). Germline and somatic mutations in meningiomas. Cancer Genet.

[19] Sievers P, Sill M, Blume C, Tauziede-Espariat A, Schrimpf D, Stichel D, Reuss DE, Dogan H, Hartmann C, Mawrin C (2021). Clear cell meningiomas are defined by a highly distinct DNA methylation profile and mutations in SMARCE1. Acta Neuropathol.

[20] Harmanci AS, Youngblood MW, Clark VE, Coskun S, Henegariu O, Duran D, Erson-Omay EZ, Kaulen LD, Lee TI, Abraham BJ, Simon M, Krischek B, Timmer M, Goldbrunner R, Omay SB, Baranoski J, Baran B, Carrion-Grant G, Bai H, Mishra-Gorur K, Schramm J, Moliterno J, Vortmeyer AO, Bilguvar K, Yasuno K, Young RA, Gunel M (2017). Integrated genomic analyses of de novo pathways underlying atypical meningiomas. Nat Commun.

[21] Reuss DE, Piro RM, Jones DT, Simon M, Ketter R, Kool M, Becker A, Sahm F, Pusch S, Meyer J (2013). Secretory meningiomas are defined by combined KLF4 K409Q and TRAF7 mutations. Acta Neuropathol.

[22] Boetto J, Apra C, Bielle F, Peyre M, Kalamarides M (2018). Selective vulnerability of the primitive meningeal layer to prenatal Smo activation for skull base meningothelial meningioma formation. Oncogene.

[23] Boetto J, Bielle F, Sanson M, Peyre M, Kalamarides M (2017). SMO mutation status defines a distinct and frequent molecular subgroup in olfactory groove meningiomas. Neuro Oncol.

[24] Smith MJ, Wallace AJ, Bennett C, Hasselblatt M, Elert-Dobkowska E, Evans LT, Hickey WF, Van Hoff J, Bauer D, Lee A (2014). Germline SMARCE1 mutations predispose to both spinal and cranial clear cell meningiomas. J Pathol.

[25] Gill CM, Loewenstern J, Rutland JW, Arib H, Pain M, Umphlett M, Kinoshita Y, McBride RB, Bederson J, Donovan M, Sebra R, Fowkes M, Shrivastava RK (2021) SWI/SNF chromatin remodeling complex alterations in meningioma*.* J Cancer Res Clin Oncol 10.1007/s00432-021-03586-7 PMC11802053 33715086

[26] Sahm F, Schrimpf D, Olar A, Koelsche C, Reuss D, Bissel J, Kratz A, Capper D, Schefzyk S, Hielscher T (2016). TERT promoter mutations and risk of recurrence in meningioma. J Natl Cancer Inst.

[27] Ueki K, Wen-Bin C, Narita Y, Asai A, Kirino T (1999). Tight association of loss of merlin expression with loss of heterozygosity at chromosome 22q in sporadic meningiomas. Cancer Res.

[28] Gabeau-Lacet D, Engler D, Gupta S, Scangas GA, Betensky RA, Barker FG, Loeffler JS, Louis DN, Mohapatra G (2009). Genomic profiling of atypical meningiomas associates gain of 1q with poor clinical outcome. J Neuropathol Exp Neurol.

[29] Suppiah S, Nassiri F, Bi WL, Dunn IF, Hanemann CO, Horbinski CM, Hashizume R, James CD, Mawrin C, Noushmehr H (2019). Molecular and translational advances in meningiomas. Neuro Oncol.

[30] Lamszus K (2004). Meningioma pathology, genetics, and biology. J Neuropathol Exp Neurol.

[31] Mawrin C, Perry A (2010). Pathological classification and molecular genetics of meningiomas. J Neurooncol.

[32] Olar A, Wani KM, Wilson CD, Zadeh G, DeMonte F, Jones DT, Pfister SM, Sulman EP, Aldape KD (2017). Global epigenetic profiling identifies methylation subgroups associated with recurrence-free survival in meningioma. Acta Neuropathol.

[33] Ma J, Hong Y, Chen W, Li D, Tian K, Wang K, Yang Y, Zhang Y, Chen Y, Song L (2020). High copy-number variation burdens in cranial meningiomas from patients with diverse clinical phenotypes characterized by hot genomic structure changes. Front Oncol.

[34] Hemmer S, Urbschat S, Oertel J, Ketter R (2019). Deletions in the 17q chromosomal region and their influence on the clonal cytogenetic evolution of recurrent meningiomas. Mol Cytogenet.

[35] Juratli TA, McCabe D, Nayyar N, Williams EA, Silverman IM, Tummala SS, Fink AL, Baig A, Martinez-Lage M, Selig MK (2018). DMD genomic deletions characterize a subset of progressive/higher-grade meningiomas with poor outcome. Acta Neuropathol.

[36] Abedalthagafi MS, Merrill PH, Bi WL, Jones RT, Listewnik ML, Ramkissoon SH, Thorner AR, Dunn IF, Beroukhim R, Alexander BM (2014). Angiomatous meningiomas have a distinct genetic profile with multiple chromosomal polysomies including polysomy of chromosome 5. Oncotarget.

[37] Lee YS, Lee YS (2020). Molecular characteristics of meningiomas. J Pathol Transl Med.

[38] Guyot A, Duchesne M, Robert S, Lia AS, Derouault P, Scaon E, Lemnos L, Salle H, Durand K, Labrousse F (2019). Analysis of CDKN2A gene alterations in recurrent and non-recurrent meningioma. J Neurooncol.

[39] Sievers P, Hielscher T, Schrimpf D, Stichel D, Reuss DE, Berghoff AS, Neidert MC, Wirsching HG, Mawrin C, Ketter R, Paulus W, Reifenberger G, Lamszus K, Westphal M, Etminan N, Ratliff M, Herold-Mende C, Pfister SM, Jones DTW, Weller M, Harter PN, Wick W, Preusser M, von Deimling A, Sahm F (2020). CDKN2A/B homozygous deletion is associated with early recurrence in meningiomas. Acta Neuropathol.

[40] Bello MJ, Aminoso C, Lopez-Marin I, Arjona D, Gonzalez-Gomez P, Alonso ME, Lomas J, de Campos JM, Kusak ME, Vaquero J, Isla A, Gutierrez M, Sarasa JL, Rey JA (2004). DNA methylation of multiple promoter-associated CpG islands in meningiomas: relationship with the allelic status at 1p and 22q. Acta Neuropathol.

[41] Shen L, Lin D, Cheng L, Tu S, Wu H, Xu W, Pan Y, Wang X, Zhang J, Shao A (2020) Is DNA methylation a ray of sunshine in predicting meningioma prognosis? Front Oncol 10 10.3389/fonc.2020.01323 PMC7498674 33014773

[42] Venza M, Visalli M, Beninati C, Catalano T, Biondo C, Teti D, Venza I (2015). Involvement of epimutations in meningioma. Brain Tumor Pathol.

[43] Di Vinci A, Brigati C, Casciano I, Banelli B, Borzì L, Forlani A, Ravetti GL, Allemanni G, Melloni I, Zona G (2012). HOXA7, 9, and 10 are methylation targets associated with aggressive behavior in meningiomas. Transl Res.

[44] Kishida Y, Natsume A, Kondo Y, Takeuchi I, An B, Okamoto Y, Shinjo K, Saito K, Ando H, Ohka F (2012). Epigenetic subclassification of meningiomas based on genome-wide DNA methylation analyses. Carcinogenesis.

[45] Ando H, Natsume A, Iwami K, Ohka F, Kuchimaru T, Kizaka-Kondoh S, Ito K, Saito K, Sugita S, Hoshino T (2013). A hypoxia-inducible factor (HIF)-3α splicing variant, HIF-3α4 impairs angiogenesis in hypervascular malignant meningiomas with epigenetically silenced HIF-3α4. Biochem Biophys Res Commun.

[46] Kandenwein JA, Park-Simon T-W, Schramm J, Simon M (2011). uPA/PAI-1 expression and uPA promoter methylation in meningiomas. J Neurooncol.

[47] Vasudevan HN, Braunstein SE, Phillips JJ, Pekmezci M, Tomlin BA, Wu A, Reis GF, Magill ST, Zhang J, Feng FY, Nicholaides T, Chang SM, Sneed PK, McDermott MW, Berger MS, Perry A, Raleigh DR (2018). Comprehensive molecular profiling identifies FOXM1 as a key transcription factor for meningioma proliferation. Cell Rep.

[48] Nassiri F, Wang JZ, Singh O, Karimi S, Dalcourt T, Ijad N, Pirouzmand N, Ng H-K, Saladino A, Pollo B (2021) Loss of H3K27me3 in meningiomas. Neuro-oncology 10.1093/neuonc/noab036 PMC8328029 33970242

[49] Katz LM, Hielscher T, Liechty B, Silverman J, Zagzag D, Sen R, Wu P, Golfinos JG, Reuss D, Neidert MC, Wirsching HG, Baumgarten P, Herold-Mende C, Wick W, Harter PN, Weller M, von Deimling A, Snuderl M, Sen C, Sahm F (2018). Loss of histone H3K27me3 identifies a subset of meningiomas with increased risk of recurrence. Acta Neuropathol.

[50] Kliese N, Gobrecht P, Pachow D, Andrae N, Wilisch-Neumann A, Kirches E, Riek-Burchardt M, Angenstein F, Reifenberger G, Riemenschneider MJ (2013). miRNA-145 is downregulated in atypical and anaplastic meningiomas and negatively regulates motility and proliferation of meningioma cells. Oncogene.

[51] Katar S, Baran O, Evran S, Cevik S, Akkaya E, Baran G, Antar V, Hanimoglu H, Kaynar MY (2017). Expression of miRNA-21, miRNA-107, miRNA-137 and miRNA-29b in meningioma. Clin Neurol Neurosurg.

[52] Goutagny S, Nault JC, Mallet M, Henin D, Rossi JZ, Kalamarides M (2014). High incidence of activating TERT promoter mutations in meningiomas undergoing malignant progression. Brain Pathol.

[53] Shankar GM, Santagata S (2017). BAP1 mutations in high-grade meningioma: implications for patient care. Neuro Oncol.

[54] Shankar GM, Abedalthagafi M, Vaubel RA, Merrill PH, Nayyar N, Gill CM, Brewster R, Bi WL, Agarwalla PK, Thorner AR, Reardon DA, Al-Mefty O, Wen PY, Alexander BM, van Hummelen P, Batchelor TT, Ligon KL, Ligon AH, Meyerson M, Dunn IF, Beroukhim R, Louis DN, Perry A, Carter SL, Giannini C, Curry WT, Cahill DP, Barker FG, Brastianos PK, Santagata S (2017). Germline and somatic BAP1 mutations in high-grade rhabdoid meningiomas. Neuro Oncol.

[55] Zador Z, Landry AP, Saha A, Cusimano MD (2020). Gene expression signatures identify biologically homogenous subgroups of grade 2 meningiomas. Front Oncol.

[56] Liu F, Qian J, Ma C (2021). MPscore: a novel predictive and prognostic scoring for progressive meningioma. Cancers.

[57] Sahm F, Schrimpf D, Stichel D, Jones DT, Hielscher T, Schefzyk S, Okonechnikov K, Koelsche C, Reuss DE, Capper D (2017). DNA methylation-based classification and grading system for meningioma: a multicentre, retrospective analysis. Lancet Oncol.

[58] Nassiri F, Mamatjan Y, Suppiah S, Badhiwala JH, Mansouri S, Karimi S, Saarela O, Poisson L, Gepfner-Tuma I, Schittenhelm J, Ng HK, Noushmehr H, Harter P, Baumgarten P, Weller M, Preusser M, Herold-Mende C, Tatagiba M, Tabatabai G, Sahm F, von Deimling A, Zadeh G, Aldape KD, M. International Consortium on (2019). DNA methylation profiling to predict recurrence risk in meningioma: development and validation of a nomogram to optimize clinical management. Neuro Oncol.

[59] Driver J, Hoffman SE, Tavakol S, Woodward E, Maury EA, Bhave V, Greenwald NF, Nassiri F, Aldape K, Zadeh G, Choudhury A, Vasudevan HN, Magill ST, Raleigh DR, Abedalthagafi M, Aizer AA, Alexander BM, Ligon KL, Reardon DA, Wen PY, Al-Mefty O, Ligon AH, Dubuc AM, Beroukhim R, Claus EB, Dunn IF, Santagata S, Bi WL (2021) A molecularly integrated grade for meningioma. Neuro-oncology 10.1093/neuonc/noab213 PMC9071299 34508644

[60] Patel AJ, Wan YW, Al-Ouran R, Revelli JP, Cardenas MF, Oneissi M, Xi L, Jalali A, Magnotti JF, Muzny DM, Doddapaneni H, Sebastian S, Heck KA, Goodman JC, Gopinath SP, Liu Z, Rao G, Plon SE, Yoshor D, Wheeler DA, Zoghbi HY, Klisch TJ (2019). Molecular profiling predicts meningioma recurrence and reveals loss of DREAM complex repression in aggressive tumors. Proc Natl Acad Sci USA.

[61] Nassiri F, Liu J, Patil V, Mamatjan Y, Wang JZ, Hugh-White R, Macklin AM, Khan S, Singh O, Karimi S, Corona RI, Liu LY, Chen CY, Chakravarthy A, Wei Q, Mehani B, Suppiah S, Gao A, Workewych AM, Tabatabai G, Boutros PC, Bader GD, de Carvalho DD, Kislinger T, Aldape K, Zadeh G (2021). A clinically applicable integrative molecular classification of meningiomas. Nature.

[62] Youngblood MW, Miyagishima DF, Jin L, Gupte T, Li C, Duran D, Montejo JD, Zhao A, Sheth A, Tyrtova E (2021). Associations of meningioma molecular subgroup and tumor recurrence. Neuro Oncol.

[63] Hong CS, Moliterno J (2020) Surgical considerations for newly diagnosed meningiomas. In: Meningiomas. Springer, pp 75–96

[64] Graillon T, Sanson M, Campello C, Idbaih A, Peyre M, Peyrière H, Basset N, Autran D, Roche C, Kalamarides M (2020). Everolimus and octreotide for patients with recurrent meningioma: results from the phase II CEVOREM trial. Clin Cancer Res.

[65] Juric V, Murphy B (2020). Cyclin-dependent kinase inhibitors in brain cancer: Current state and future directions. Cancer Drug Resist.

[66] He S, Pham MH, Pease M, Zada G, Giannotta SL, Wang K, Mack WJ (2013). A review of epigenetic and gene expression alterations associated with intracranial meningiomas. Neurosurg Focus.

[67] Galani V, Lampri E, Varouktsi A, Alexiou G, Mitselou A, Kyritsis AP (2017). Genetic and epigenetic alterations in meningiomas. Clin Neurol Neurosurg.

[68] San-Miguel T, Navarro L, Megias J, Munoz-Hidalgo L, Gil-Benso R, Roldan P, Lopez-Gines C, Cerda-Nicolas M (2019). Epigenetic changes underlie the aggressiveness of histologically benign meningiomas that recur. Hum Pathol.

[69] Samal S, Patnaik A, Sahu F, Purkait S (2020). Altered expression of epigenetic modifiers EZH2, H3K27me3, and DNA methyltransferases in meningiomas - prognostic biomarkers for routine practice. Folia Neuropathol.

[70] Xing H, Wang S, Li Q, Ma Y, Sun P (2018). Long noncoding RNA LINC00460 targets miR-539/MMP-9 to promote meningioma progression and metastasis. Biomed Pharmacother.

[71] Li T, Ren J, Ma J, Wu J, Zhang R, Yuan H, Han X (2019). LINC00702/miR-4652–3p/ZEB1 axis promotes the progression of malignant meningioma through activating Wnt/beta-catenin pathway. Biomed Pharmacother.

[72] Zhang Y, Yu R, Li Q, Li Y, Xuan T, Cao S, Zheng J (2020). SNHG1/miR-556-5p/TCF12 feedback loop enhances the tumorigenesis of meningioma through Wnt signaling pathway. J Cell Biochem.

[73] Balik V, Srovnal J, Sulla I, Kalita O, Foltanova T, Vaverka M, Hrabalek L, Hajduch M (2013). MEG3: a novel long noncoding potentially tumour-suppressing RNA in meningiomas. J Neurooncol.

[74] Huntoon K, Toland AMS, Dahiya S (2020). Meningioma: a review of clinicopathological and molecular aspects. Front Oncol.

[75] Nassiri F, Wang JZ, Singh O, Karimi S, Dalcourt T, Ijad N, Pirouzmand N, Ng HK, Saladino A, Pollo B, Dimeco F, Yip S, Gao A, Aldape KD, Zadeh G, M. International Consortium on (2021). Loss of H3K27me3 in meningiomas. Neuro Oncol.

[76] Olivera M, Martinez C, Molina JA, Alonso-Navarro H, Jimenez-Jimenez FJ, Garcia-Martin E, Benitez J, Agundez JA (2006). Increased frequency of rapid acetylator genotypes in patients with brain astrocytoma and meningioma. Acta Neurol Scand.

[77] Juratli TA, McCabe D, Nayyar N, Williams EA, Silverman IM, Tummala SS, Fink AL, Baig A, Martinez-Lage M, Selig MK, Bihun IV, Shankar GM, Penson T, Lastrapes M, Daubner D, Meinhardt M, Hennig S, Kaplan AB, Fujio S, Kuter BM, Bertalan MS, Miller JJ, Batten JM, Ely HA, Christiansen J, Baretton GB, Stemmer-Rachamimov AO, Santagata S, Rivera MN, Barker FG, Schackert G, Wakimoto H, Iafrate AJ, Carter SL, Cahill DP, Brastianos PK (2018). DMD genomic deletions characterize a subset of progressive/higher-grade meningiomas with poor outcome. Acta Neuropathol.

[78] Liu J, Xia C, Wang G (2020). Multi-omics analysis in initiation and progression of meningiomas: from pathogenesis to diagnosis. Front Oncol.

[79] Weber RG et al (1997) Analysis of genomic alterations in benign, atypical, and anaplastic meningiomas: toward a genetic model of meningioma progression. Proc Nat Acad Sci 94(26):14719–14724 10.1073/pnas.94.26.14719 PMC25103 9405679

