# Correction to: The integrated multiomic diagnosis of sporadic meningiomas: a review of its clinical implications

**DOI:** 10.1007/s11060-021-03937-x

**Published:** 2021-12-30

**Authors:** Stephanie M. Robert, Shaurey Vetsa, Arushii Nadar, Sagar Vasandani, Mark W. Youngblood, Evan Gorelick, Lan Jin, Neelan Marianayagam, E Zeynep Erson-Omay, Murat Günel, Jennifer Moliterno

**Affiliations:** 1 Department of Neurosurgery, Yale School of Medicine, 15 York St, LLCI 810, New Haven, CT 06520-8082 USA; 2 The Chenevert Family Brain Tumor Center, Smilow Cancer Hospital, New Haven, CT USA; 3 Department of Neurological Surgery, Northwestern University, Chicago, IL USA

## Correction to: Journal of Neuro-Oncology 10.1007/s11060-021-03874-9

In this article the author name Neelan Marianayagam was incorrectly written as Neelan Marianayanam. The original article has been corrected.

